# Ensuring access to novel COVID-19 therapeutics in Pacific island countries and areas

**DOI:** 10.5365/wpsar.2023.14.2.1000

**Published:** 2023-06-22

**Authors:** Gereltuya Dorj, Eva Mata Martinez, Karen Hammad, Biniam Getachew Kabethymer, Nuha Mahmoud

**Affiliations:** a World Health Organization Division of Pacific Technical Support, Suva, Fiji.; b Quality Use of Medicines and Pharmacy Research Centre, University of South Australia, Adelaide, South Australia, Australia.; c Menzies Health Institute Queensland, Griffith University, Nathan, Queensland, Australia.; d College of Nursing and Health Sciences, Flinders University, Adelaide, South Australia, Australia.

## Abstract

**Problem:**

As of November 2022, over 417 397 confirmed cases and 2631 deaths related to coronavirus disease (COVID-19) were reported in Pacific island countries and areas (PICs). Most PICs have faced challenges accessing therapeutics recommended for the treatment of COVID-19 due to their high demand worldwide and supply chain constraints.

**Context:**

The World Health Organization (WHO) coordinates and provides tailored technical and operational support to 21 PICs. Since the start of the pandemic, WHO has worked with partners to establish a mechanism to ensure equitable access to three novel COVID-19 therapeutics (tocilizumab, molnupiravir and nirmatrelvir/ritonavir) for lower-income countries, including 11 eligible PICs.

**Action:**

WHO coordinated the requests, procurement and distribution of the three novel therapeutics. In addition, WHO supported PICs by providing trainings in clinical management of COVID-19, developing critical supply needs estimates, and facilitating regulatory approval of clinical therapeutics, including emergency use authorization.

**Lessons learned:**

The main barriers to procurement of novel COVID-19 therapeutics were identified as prolonged negotiations with licence holders, sourcing funding, the high cost of therapeutics and limited capacity to provide safety monitoring.

**Discussion:**

Uninterrupted supply and availability of essential medicines in the Pacific region is dependent on external and local sourcing. To overcome procurement barriers and ensure access to novel COVID-19 therapeutics in PICs, WHO‘s pandemic support to Member States focused on strengthening regulatory requirements, safety monitoring and supply chain activities.

The first case of coronavirus disease (COVID-19) in the Pacific was reported in March 2020 in French Polynesia. (*1*) Since then, a total of 417 397 cases and 2631 deaths have been reported across the Pacific (data as of mid-November 2022). (*1*) Among the Pacific island countries and areas (PICs), Nauru has had the highest incidence rate, with 42 551 cumulative cases per 100 000 population. (*1*)

Several novel therapeutics for the treatment of patients with COVID-19 have been recommended by the World Health Organization (WHO). (*2*, *3*) While many high-income countries have the resources to procure and implement pharmaceutical interventions, most PICs have faced difficulties in accessing and delivering COVID-19 therapeutics to their populations, largely due to high worldwide demand and supply chain constraints. This report describes the challenges experienced by WHO and partnering organizations at national and local levels in relation to ensuring access to novel COVID-19 therapeutics in PICs and the progress that has been made in overcoming those challenges.

## CONTEXT

**Fig. 1:**
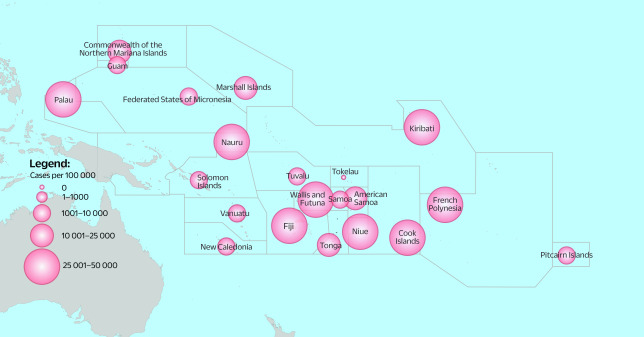
Fig. 1. Map of Pacific island countries and areas, by corresponding cumulative COVID-19 cases per 100 000 population

The WHO Division of Pacific Technical Support (DPS) coordinates and provides tailored technical and operational support to 21 PICs (**Fig. 1**), which collectively are home to 3.2 million people spread across an ocean that covers 30% of Earth’s surface. (*4*) According to the World Bank, French Polynesia, New Caledonia and the Commonwealth of the Northern Mariana Islands (CNMI) are categorized as high-income nations, while American Samoa, Kiribati, the Federated States of Micronesia (FSM), Samoa, Solomon Islands and Vanuatu are ranked as low-income countries. The remaining countries and areas are classified as upper-middle-income nations. (*5*)

Owing to their limited resources, dependence on international trade, remote location and fragile ecosystems, PICs are highly susceptible to the threats to national and regional health security posed by emerging and re-emerging infectious diseases and climate change. (*4*) In addition, the Pacific region is prone to natural disasters such as floods, cyclones and volcanic eruptions that can disrupt health systems. Although the geographical remoteness of PICs provides some advantages in isolating and preventing transmission of infectious disease outbreaks, few escaped the impacts of the COVID-19 pandemic. Fiji, French Polynesia, Guam, FSM and New Caledonia all experienced outbreaks of widespread community transmission due to the Delta and Omicron variants. (*1*)

[**insert** Figure 1]

Pharmaceutical interventions such as vaccines and therapeutics have proven effective against COVID-19 and are a vital part of national strategies to prevent SARS-CoV-2 from circulating and threatening health and economic security. However, COVID-19 therapeutics are subject to stringent approval processes by regulatory authorities, such as WHO, the United States (US) Food and Drug Administration (FDA), the European Medicines Agency (EMA), the Therapeutic Goods Administration of Australia, as well as the New Zealand Medicines and Medical Devices and Safety Authority (MEDSAFE). At the time of writing, only tocilizumab, molnupiravir and nirmatrelvir/ritonavir were available for WHO procurement under the emergency use listing. (*6*) However, other authorities have approved the use of alternative therapeutics for COVID-19 such as sotrovimab, casirivimab/imdevimab, cilgavimab/tixagevimab and tofacitinib in some PICs.

During 2020 and 2021, WHO, together with partners, responded to the surge in COVID-19 cases in the Pacific by assisting PICs in accessing essential medicines such as dexamethasone and heparin, as well as oxygen. (*2*, *3*) In 2022, the three novel therapeutics recommended in WHO’s Therapeutics and COVID-19: Living Guideline (*2*) (tocilizumab for hospitalized patients, and molnupiravir and nirmatrelvir/ritonavir for non-severe cases) were also made available through WHO’s Access to COVID-19 Tools (ACT) Accelerator, a global mechanism that ensures appropriate allocation and equitable distribution of limited supplies of expensive novel COVID-19 therapeutics. (*6*)

## ACTION

Novel COVID-19 therapeutics need to be properly regulated and distributed as prescription medication with appropriate information provided to health-care workers and patients to minimize potential adverse events. (*2*) During 2020–2022, WHO DPS supported its Pacific Member States in accessing and distributing recommended novel COVID-19 therapeutics of assured quality by:

coordinating requests, procurement and distribution of tocilizumab, molnupiravir and nirmatrelvir/ritonavir through the ACT-Accelerator;
developing and updating clinical management guidelines and standard operating procedures and delivering trainings in the clinical management of COVID-19;
facilitating regulatory approval of COVID-19 therapeutics, including emergency use authorization; and
developing estimates of critical supply needs.

### Coordination of requests, procurement and distribution

As COVID-19 spread globally, many PICs mounted a multisectoral response to the pandemic – introducing border closures, mandatory isolation, and quarantine for suspected and confirmed cases – in a bid to contain cases to one geographical cluster and to buy time until pharmaceutical interventions could be implemented. PICs have had access to tocilizumab since May 2022 and molnupiravir since November 2022. Eight PICs have recently accepted allocations of nirmatrelvir/ritonavir.

During 2022, WHO DPS received requests for COVID-19 therapeutics from the 11 PICs that were eligible for support through the WHO ACT-Accelerator platform. By October 2022, WHO had procured 1155 doses of tocilizumab injections at an estimated cost of US$ 317 900 to PICs (**Table 1**), and six countries were on track to take delivery of their allocated 2016 courses of molnupiravir. In November 2022, eight PICs opted-in to access 2736 courses of nirmatrelvir/ritonavir; procurement has entered the distribution phase with delivery carried out in March–May 2023. Some PICs (American Samoa, the Marshall Islands, FSM and Palau) were able to access COVID-19 therapeutics in early 2022 through the support of other partners including the US Centers for Disease Control and Prevention.

**Table 1:** Procurement and supply of COVID-19 therapeutics in 11 Pacific island countries and areas by October 2022

Country or area	Tocilizumab (vials)	Molnupiravir (courses)	Nirmatrelvir/ritonavir (courses)
American Samoa	105	NA	NA
Fiji	105	360	96
Kiribati	105	432	576
Marshall Islands	105	216	240
Federated States of Micronesia (Federated States of)	105	NA	336
Nauru	105	NA	192
Samoa	105	432	384
Solomon Islands	105	NA	NA
Tonga	105	NA	240
Tuvalu	105	72	672
Vanuatu	105	504	NA
**Total**	**1155**	**2016**	**2736**

NA: not applicable.

### Clinical management support

As part of its pandemic support to PICs, WHO DPS developed and updated treatment algorithms and standard operating procedures for the clinical management of COVID-19. Trainings in clinical management, prescription and administration of novel therapeutics were delivered via in-person deployments and face-to-face trainings for health managers and clinicians across the PICs.

Between July and October 2022, four webinar sessions on the implementation of COVID-19 therapeutics contextualized for clinical practice in the PICs were delivered via Zoom (Zoom Video Communications, Inc., San Jose, CA, USA). The webinars covered topics such as indications for use, storage conditions, care pathways, therapeutic management of severe and non-severe cases, and safe and appropriate use, as well as country experiences. Trainers comprised experts and clinicians from WHO DPS, the WHO Regional Office for the Western Pacific, the Australian Therapeutic Goods Administration, the Central and Northern Adelaide Local Health Networks (Adelaide, Australia) the Royal Alfred Hospital (Melbourne, Australia), the University of South Australia (Adelaide, Australia), New Zealand MEDSAFE, the WHO Global Outbreak Alert and Response Network, Fiji, the Marshall Islands, FSM and Palau. More than 150 health-care professionals including nurses, medical doctors, pharmacists and health advisers attended the webinars. Attendees represented 12 PICs – Fiji, Kiribati, the Marshall Islands, FSM, Nauru, Niue, Papua New Guinea, Solomon Islands, Tokelau, Tonga, Tuvalu and Vanuatu. The webinars provided a platform not only for participants to learn lessons from countries such as Australia and New Zealand (who were also represented among the attendees), but also for neighbouring countries to share information and their experiences of therapeutics and clinical management of COVID-19 cases.

### Regulatory approvals

In the Pacific, the level of regulatory systems development for medicines is either non-existent or very limited. (*7*) Many PICs have a legal basis for pharmaceutical activities, such as registration of medicines, regulation and control of dangerous drugs and poisons, licensing of establishments, regulation of the pharmacy profession and reporting of adverse events. However, in many cases, the existence of national legislation does not necessarily translate into implementation and enforcement. The main barriers are a lack of human and technical capacity, as well as limited financial resources of the regulatory authorities. (*7*)

WHO’s pandemic response thus included facilitating regulatory approval of COVID-19 therapeutics in PICs with limited technical capacity and resources. In addition to conducting literature reviews and disseminating emerging evidence on novel therapeutics, WHO assisted countries in navigating the necessary regulatory processes and systems that precede the approval of novel medical products by the relevant regulatory authorities, including product registration and licensing, and post-marketing surveillance activities. Based on the information and support provided by WHO, regulatory approvals – in the form of emergency use authorizations and adaptive licensing mechanisms – were issued for COVID-19 therapeutics by the relevant stakeholders, on average within 4 weeks of the initial request. In the majority of cases, approvals were granted by established regulatory authorities and mechanisms such as prequalification by WHO, US FDA, (*8*) EMA, (*9*) the Australian Therapeutic Goods Administration (*10*) and New Zealand MEDSAFE. (*11*)

### Critical supply estimates for COVID-19 therapeutics

To forecast the quantity of essential medicines and therapeutics required to treat COVID-19 cases in PICs, WHO DPS developed a series of critical supply estimates. Initially, these supply estimates were based on a single COVID-19 wave and on current treatment guidelines. (*2*) Estimates thus allowed for the treatment with oral antivirals of those non-severe cases at increased risk for severe disease. As molnupiravir and nirmatrelvir/ritonavir have the same target population, the demand for these therapeutics was assumed to be the same to avoid double-counting.

WHO DPS’s critical supply estimates were used as the basis for expressions of interest or requests for COVID-19 therapeutics by PICs eligible for support through WHO’s ACT-Accelerator. Such requests require countries to submit information on important factors such as minimum amount to meet their needs, time period, supply availability and prioritization criteria. (*6*) The final allocation of COVID-19 therapeutics was performed by WHO in partnership with Wellcome and Tous Unis pour Aider (UNITAID), (*6*) and was based on (a) demand, (b) the epidemiological situation in each country and (c) the global supply of therapeutics for populations in low- and middle-income countries. The allocation mechanism also considered treatment goals, the target population (according to WHO treatment guidelines) and rate-limiting criteria as appropriate.

## LESSONS LEARNED

Securing access to novel COVID-19 therapeutics in the PICs has been challenging for several reasons. The limited availability of evidence for the use of novel therapeutics was an early problem not just in PICs but around the globe. In the PICs, a lack of local capacity for developing national guidelines and standard operating documents governing the use of novel therapeutics was a major barrier to early implementation; other challenges have included the lengthy and protracted nature of procurement negotiations with manufacturers, licence holders and funding sources, and the high cost of therapeutics. Significant mark-up costs, other additional charges, and the costs associated with logistics and transportation of therapeutics were all factors that contributed to the high cost of therapeutics for PICs. Recognizing these key barriers to access, WHO DPS support to PICs was targeted at identifying funding partners and facilitating discussions between suppliers and counterparts across the Pacific. Moreover, procurement of COVID-19 therapeutics through the WHO ACT-Accelerator enabled high-cost medicines to be supplied to eligible PICs at an affordable cost.

In terms of the distribution of novel COVID-19 therapeutics within countries, several important lessons were learned. Several PICs, notably the US-affiliated Pacific islands including the Marshall Islands and FSM, set up community-based “test-to-treat” centres where patients could be tested and, if found positive, could be prescribed an oral antiviral straightaway (if they were eligible for antiviral treatment, i.e. in a high-risk category for severe COVID-19 disease). (*12*) The success of this “one-stop-shop” test-to-treat initiative suggests that similar strategies could be adopted and implemented across the Pacific.

WHO-recommended treatments for severe or critical COVID-19 infection, which include interleukin-6 receptor blockers (tocilizumab or sarilumab) and corticosteroids, (*2*, *3*) were implemented in PICs, as elsewhere, in clinical settings only. However, when evidence emerged that patients treated with corticosteroids who were coinfected with *Strongyloides stercoralis*, (*13*) an intestinal roundworm, were at higher risk of developing hyperinfection, (*14*) it became apparent that specific guidance for the treatment of severe COVID-19 in the Pacific was needed, especially in those tropical and subtropical PICs where *Strongyloides stercoralis* is prevalent. WHO continues to provide support to address specific clinical issues common in the Pacific.

Several PICs experienced difficulties in procuring an adequate supply of some essential medicines required for treatment of moderate-to-severe COVID-19, in particular dexamethasone, (*2*, *3*) which is not manufactured in the Pacific. Shortages in other essential drugs were also reported, including saline solution, which is needed to dilute medicines such as tocilizumab. (*3*) Alongside delivering ongoing technical support, WHO DPS also successfully procured and supplied dexamethasone injections to 10 Member States in response to emergency requests.

In addition to supply chain issues, the COVID-19 pandemic highlighted the limited capacity of PICs to conduct safety monitoring activities for novel therapeutics. Although existing data suggest that newly introduced COVID-19 therapeutics were generally well tolerated, (*2*) the effects of long-term use have yet to be studied, and thus there is a need for ongoing safety monitoring. It was also noted that while several PICs had existing legal provision for monitoring adverse drug events and adverse events following immunization, none had a robust pharmacovigilance system in place before the pandemic. (*15*) Historically, reporting was undertaken at the health service provider level but was not routinely shared nationally or with other countries. (*15*) During the pandemic phase, WHO DPS assessed PICs’ needs and demands for establishing and strengthening post-marketing surveillance systems and provided continuous technical guidance and trainings. Supported by WHO DPS, Fiji has recently renewed its full membership in the WHO Programme for International Drug Monitoring. Members of this programme work nationally and collaborate internationally to monitor and identify any potential medicine-induced harms.

Finally, the pandemic phase focused attention on the potential risk to public health posed by the general absence of robust quality control and assurance systems for medical products that exist across the Pacific. The identification of batches of falsified COVID-19 therapeutics in countries of neighbouring regions (*16*) in particular highlighted the lack of sufficient laboratory testing capacity in many PICs. While countries such as the Cook Islands, Fiji, Kiribati, the Marshall Islands, FSM, Nauru, Palau, Papua New Guinea, Tonga, Tuvalu and Vanuatu have laws that mandate the regulation of medical products, (*15*) during the pandemic most relied on the quality supply chain of wholesalers or the limited laboratory support provided by WHO to ensure that their COVID-19 therapeutics were safe, effective and of assured quality. (*15*) Going forward, WHO plans to extend its laboratory support to PICs by providing a wider range of laboratory tools, technical advice and training. For example, quality assurance trainings are scheduled to take place in Solomon Islands.

### Limitations

This report has some inevitable limitations. As it describes the experience of PICs, due to the huge diversity in social systems, health-care provision, economic factors and geography, the findings might not be generalizable to all countries in the Pacific. Likewise, each PIC is unique in its culture and customs, and therefore even within the group of PICs, the responses and lessons learned may not apply universally. Nevertheless, in a field with a paucity of literature, this report not only contributes to new knowledge but also provides some important lessons for countries that share similar characteristics, namely geographical isolation and limited regulatory systems, in terms of the management of future infectious disease outbreaks in which novel therapeutic interventions are required.

## Conclusion

Throughout the COVID-19 pandemic, WHO DPS, in collaboration with partners, has delivered tailored support to PICs. This support has taken the form of assistance with procurement and emergency use authorization of novel therapeutics; provision of clinical management guidance and technical support; and regulatory system strengthening, in particular building capacity in safety monitoring and quality assurance programmes. Looking ahead, WHO DPS support should continue to be focused on strengthening regulatory requirements, safety monitoring and supply chain activities to ensure access to and implementation of novel COVID-19 therapeutics in all PICs. To ensure sustainable access to quality-assured therapeutics in the event of future pandemics, it will be important to continue to develop methodologies to estimate critical supply needs and demands.
